# Update on the Phylodynamics of SADS-CoV

**DOI:** 10.3390/life11080820

**Published:** 2021-08-11

**Authors:** Fabio Scarpa, Daria Sanna, Ilenia Azzena, Piero Cossu, Marta Giovanetti, Domenico Benvenuto, Elisabetta Coradduzza, Ivailo Alexiev, Marco Casu, Pier Luigi Fiori, Massimo Ciccozzi

**Affiliations:** 1 Department of Veterinary Medicine, University of Sassari, 07100 Sassari, Italy; iazzena@uniss.it (I.A.); picossu@uniss.it (P.C.); marcasu@uniss.it (M.C.); 2 Department of Biomedical Sciences, University of Sassari, 07100 Sassari, Italy; darsanna@uniss.it (D.S.); fioripl@uniss.it (P.L.F.); 3 Flavivirus Laboratory, Oswaldo Cruz Institute, Oswaldo Cruz Foundation, Rio de Janeiro 21040-360, Brazil; marta.giovanetti@ioc.fiocruz.br; 4 Unit of Medical Statistics and Molecular Epidemiology, University Campus Bio-Medico of Rome, 00128 Rome, Italy; domenicobenvenuto95@gmail.com (D.B.); m.ciccozzi@unicampus.it (M.C.); 5 Istituto Zooprofilattico Sperimentale della Sardegna, Sassari, Italy; elisabetta.coradduzza@izs-sardegna.it; 6 National Reference Laboratory of HIV, National Center of Infectious and Parasitic Diseases, 1504 Sofia, Bulgaria; ivoalexiev@yahoo.com

**Keywords:** phylodynamics, genetic diversity, coronavirus, epidemiology, *Alphacoronavirus*

## Abstract

Coronaviruses are known to be harmful and heterogeneous viruses, able to infect a large number of hosts. Among them, SADS-CoV (Swine Acute Diarrhea Syndrome Coronavirus), also known as PEAV (Porcine Enteric Alphacoronavirus), or SeA-CoV (Swine Enteric Alphacoronavirus), is the most recent Alphacoronavirus discovered, and caused several outbreaks reported in Chinese swine herds between late 2016 and 2019. We performed an upgraded phylodinamic reconstruction of SADS-CoV based on all whole genomes available on 21 June 2021. Results showed a very close relationship between SADS-CoV and HKU2-like CoV, which may represent the evolutionary intermediate step towards the present SADS-CoV. The direct progenitor of SADS-CoV is so far unknown and, although it is well known that horseshoe bats are reservoirs for *Rhinolophus bat coronavirus HKU2-like* (HKU2-like CoVs), the transmission path from bats to pigs is still unclear. The discrepancies in the phylogenetic position of rodent CoV, when different molecular markers were considered, corroborate the recombination hypothesis, suggesting that wild rats, which are frequent in farms, may have played a key role. The failure of the attempt at molecular dating, due to the lack of a clock signal, also corroborates the occurrence of a recombination event hypothesis. Zoonotic infections originating in wildlife can easily become a significant threat for human health. In such a context, due to the high recombination and cross-species capabilities of Coronavirus, SADS-CoV represents a possible high-risk pathogen for humans which needs a constant molecular monitoring.

## 1. Introduction

Coronaviruses are enveloped single positive-stranded RNA viruses belonging to the family Coronaviridae, that have been isolated in several avian [1,2] and a few mammal hosts [3]. Their genome is the largest among those of single-stranded RNA viruses [4], ranging from 26 to 32 kbases in length [5]. The genome of coronaviruses includes several open reading frames (ORFs) (from 6 to 11) [6]. The largest ORF, which represents about 67% of whole viral genome, encodes 16 non-structural proteins (nsps), while the remaining ORFs encode accessory and structural proteins [4]. The main structural proteins are the spike surface glycoprotein (S), small envelope protein I, membrane protein (M), and nucleocapsid protein (N). The whole family Coronaviridae is represented by four genera, i.e., *Alphacoronavirus*, *Betacoronavirus*, *Gammacoronavirus*, and *Deltacoronavirus* (see, i.a., [7] and references therein).

**Figure 1:**
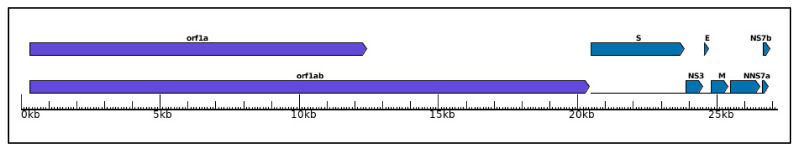
Genomic structure of SADS-CoV (Accession number: MT294722). The image was obtained by the Genome Image Map tool implemented in ViPR (Virus Pathogen Resources) (available at https://www.viprbrc.org/, accessed on 23 June 2021) and subsequently edited using the software GIMP (available at https://www.gimp.org, accessed on 23 June 2021).

Among them, the genus *Alphacoronavirus*, which was previously known as a coronavirus belonging to the “phylogroup 1” (see, e.g., [8]), presents a high level of heterogeneity for what concerns the potential for infecting different hosts [9]. As also reported for *Betacoronavirus*, the lineages of *Alphacoronavirus* descend from the bat viral gene pool [10]. The most recently discovered *Alphacoronavirus* is SADS-CoV, also known as PEAV (Porcine Enteric Alphacoronavirus) [11] and SeA-CoV (Swine Enteric Alphacoronavirus) [12], which has been isolated for the first time in early 2017 in the Chinese province of Guangdong. SADS-CoV was the cause of the regional outbreaks in southern region of China [11,12,13] and produces in infected hosts the typical symptoms of other swine coronaviruses (i.e., PEDV—porcine epidemic diarrhoea virus, PRCV—porcine respiratory coronavirus, TGEV—transmissible gastroenteritis virus, PHEV—porcine hemagglutinating encephalomyelitis virus, and PDCOV—porcine deltacoronavirus), such as severe diarrhea, vomiting, and dehydration [12], with a 35% mortality rate in piglets that are less than 10 days old [13]. Pan et al. [12] reported the whole genome of the first SADS-CoV (i.e., MF370205), which shows a composition equal to other *Alphacoronavirus*, with ORFs encoding for four structural proteins (S, E, M, and N), an accessory ORF3 between S and E, and two overlapping ORFs (NS7a and NS7b) positioned after the gene N (see Figure 1 for details).

SADS-CoV, similar to what occurred for SARS-CoV (Severe Acute Respiratory Syndrome Coronavirus), originated in bats belonging to the genus *Rhinolophus* (horseshoe bats), and since its first finding has shown a close phylogenetic relationship with the species *Rhinolophus bat coronavirus HKU2* [12]. The first SADS-CoV outbreak started in early 2017 in the South-East of China (province of Guangdong) and spread rapidly in several farms, persisting for few months. In May of the same year, the epidemic was under control, and an acquiesce period started, during which the virus apparently vanished from pig herds (see [13,14], with the only exception for the occurrence of a new strain found in pig farms placed in the province of Fujian (located North of the province of Guangdong) (see [15,16]). After two years from its first appearance, in early 2019, SADS-CoV re-emerged on a large scale, with about 2,000 piglets dead of diarrhea in a single farm [14]. This occurrence evidences the importance to perform constant molecular surveys to monitor the population dynamics of the virus, in order to protect livestock and human health. The pandemic emergency of SARS-CoV2 showed how dangerous coronaviruses can became when a homologous recombination happens between different viruses, causing cross-species transmission [17]. In such a context, recent surveys on coronaviruses isolated in bats demonstrated that alphacoronaviruses have higher odds to perform host-switching than betacoronaviruses (also across less phylogenetically related host taxa), which are more highly constrained by phylogenetic distance among hosts [18].

Here, we performed an upgraded phylodinamic reconstruction of SADS-CoV based on all whole genomes available in NCBI-Virus and GenBank database to date (last update 21 June 2021), for which information on sampling date are present. The research aimed to provide an upgrade of the knowledge on population dynamic of this virus, considering that zoonotic coronaviruses are known to be an ongoing threat which require a continuous molecular monitoring (see, e.g., [14]), as zoonotic infections originating in wildlife can become a significant threat to human health [19].

## 2. Materials and Methods

### 2.1. Datasets

The analyses were based on two datasets. The first dataset was composed by 162 whole genomes of *Alphacoronavirus*, 48 of which belonged to SADS-CoV, available on 21 June 2021, sampled between 2016 and 2019. The remaining genomes belonged to the most representative groups of the genus *Alphacoronavirus*, i.e., camel, Human 229E and NL63, PEDV, PRCV, TGEV, mink, canine, feline, HKU2, and Rodent alphacoronavirus. The second dataset, including the same viral specimens, only considered the gene S. All genomes were taken from NCBI Virus database (https://www.ncbi.nlm.nih.gov/labs/virus/vssi/#/, accessed on 21 June 2021). 

### 2.2. Phylodynamic Analyses

Genomes were aligned by using the algorithm L-INS-I implemented in Mafft 7.471 [20] under the preconfigured alignment strategy that favors accuracy. The best probabilistic model of genome evolution was estimated by means of jModeltest 2.1.1 [21], with a maximum likelihood optimized search, using both the Akaike Information Criterion (AIC), and the Bayesian Information Criterion (BIC). Phylogenetic relationship among viral genomes was investigated using Bayesian Inference (BI), which was carried out using the software MrBayes 3.2.7 [22] specifying the evolutionary model obtained by jModeltest. Two independent runs, each consisting of four Metropolis-coupled MCMC chains (one cold and three heated chains), were run simultaneously for 5,000,000 generations, sampling trees every 1000 generations. The first 25% of the 10,000 sampled trees was discarded as burn-in. Runs were checked for the convergence of chains ensuring that the Average Standard Deviation of Split Frequencies (ASDSF) approached 0 [22], and the Potential Scale Reduction Factor (PSRF) was around 1 [23], by following Scarpa et al. [24]. The phylogenetic trees were edited and visualized using FigTree 1.4.4 (available at http://tree.bio.ed.ac.uk/software/figtree/, accessed on 26 June 2021). The same analyses, with the same options, were also performed for the second dataset based on gene S.

A subset including only whole genomes of SADS-CoV, HKU2, and HKU2-like (*n* = 52), for which sampling date was available, was analysed in order to co-estimate the evolutionary rate and the dated tree were by using a Bayesian Monte Carlo Markov Chain (MCMC) approach by means of the software Beast 1.10.4 [25] implementing the evolutionary model previously selected. All parametric demographic models of population growth and piecewise-constant models were compared under both a strict and an uncorrelated log-normal relaxed clock model. Demographic and clock models have been selected, choosing the higher value of natural logarithm of Bayes Factor multiplied by two (2lnBF), using Tracer 1.7 [26] following Kass and Raftery [27] with the modifications proposed by Suchard et al. [28]. After the selection of the better model, runs up to 500 million generations were performed. The resulting log files were examined using the software Tracer 1.7 [26]. In order to investigate the temporal signal and ‘clock-likeness’ of molecular phylogenies, we performed a tip-to-root regression by using the software TempEst 1.5.3 [29]. The tree used for the analysis in TempEst, which must not be inferred under a molecular-clock assumption, was obtained by using the software IQ-Tree [30] under the nucleotide substitution model inferred in jModelTest. 

All runs were executed by means of the Cipres Phylogenetic Portal [31].

## 3. Results

**Figure 2:**
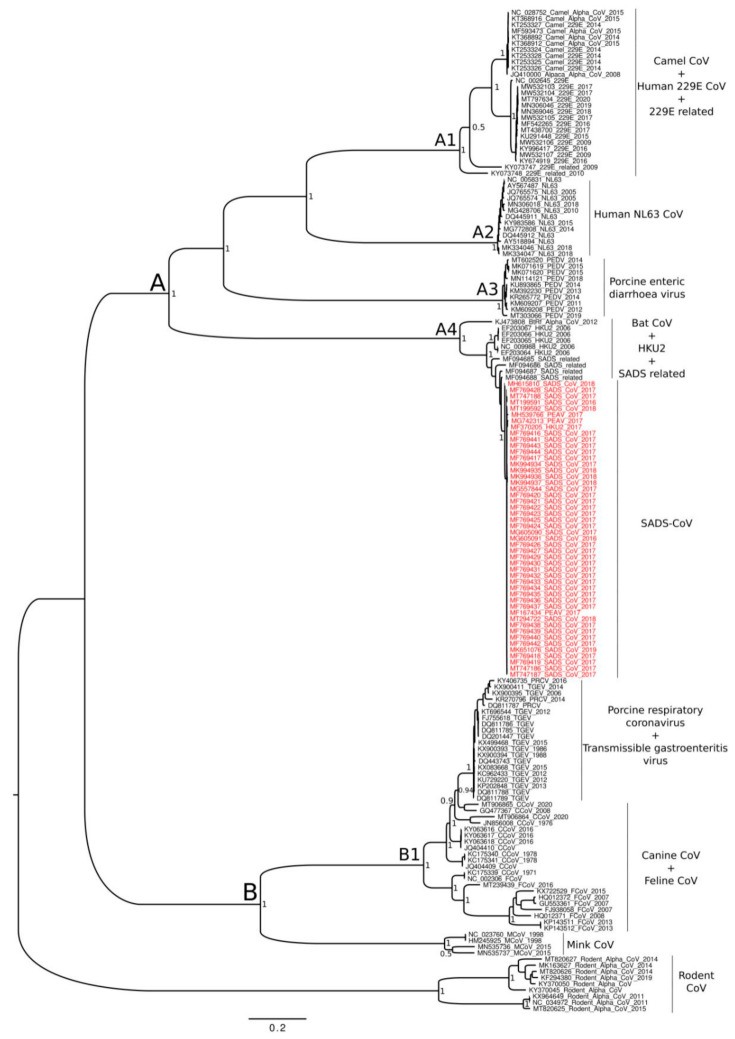
Bayesian phylogenetic tree based on whole genomes. Values of node supports are expressed as Posterior Probabilities. SADS-CoV genomes are in red font.

The Bayesian phylogenetic tree estimated on the dataset including 162 whole genomes of *Alphacoronavirus* (Figure 2) shows fully supported clades (posterior probabilities = 1) only. Overall, the tree shows a clear and statically significant genetic structuring which is consistent with the viral strains taxonomic classification. After applying a midpoint-rooting, the clade composed by rodent CoV set as an outgroup, externally to the large group including the remaining *Alphacoronavirus* which are separated by a dichotomy in clades A and B. In the clade A, four main sub-clades occur (A1, A2, A3, and A4). In particular, within the sub-clade A1, camel CoV genomes represent the sister group with the major cluster of human alphacoronavirus 229E, which is likely polyphyletic, as two sequences are placed externally to this main dichotomy. The sub-clade A2 groups human alphacoronavirus NL63 and is the sister group of A1. The sub-clade A3 includes only genomes of PEDV-CoV and represents the sister group of the cluster A1 + A2. In the sub-clade A4, the genomes of SADS-CoV clustered all together and their clade groups with SADS-CoV related (also called HKU2-like). The clade containing SADS-CoV + SADS-CoV related shows, in turn, a sister group relationship with *Rhinolophus bat coronavirus HKU2*. Among the sub-clades within the clade A, the A4 is the most external.

Within the clade B, the subclade B1 groups genomes belonging to Transmissible gastroenteritis coronavirus (TGEV) together with canine and feline alphacoronavirus. A cluster external to B1 and represented by genomes of Mink CoV, is also included within the clade B.

**Figure 3:**
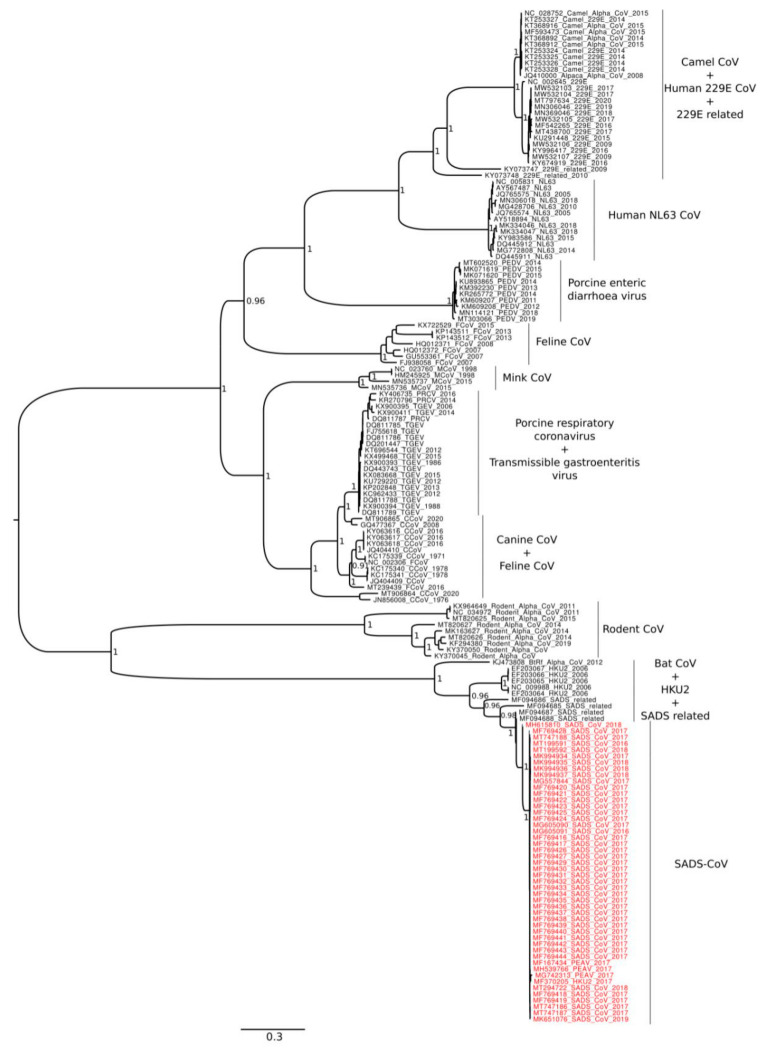
Bayesian phylogenetic tree based on the gene S. Values of node supports are expressed as Posterior Probabilities. SADS-CoV sequences are in red font. Accession numbers are the same showed in Figure 2.

The Bayesian phylogenetic tree, which was performed for the S gene based on the same dataset of *Alphacoronavirus* (*n* = 162), shows two main clades (Figure 3). The first one is composed by camel, 229E, NL63, PEDV, feline, mink, TGEV, and canine alphacoronavirus, and the second is composed by *Rhinolophus bat coronavirus HKU2*, rodent, and SADS-CoV.

Regarding the dating analyses, posterior tests on Beast results indicated the lack of a clock signal. Results were confirmed by the tip-to-root regression, which depicted very little correlation between the genetic divergence and the sampling date with CC (coefficient correlation): 0.2 and R^2^ (coefficient of determination): 5.9 × 10^−2^. This condition can be explained with the occurrence of some factors disturbing the molecular clock. Indeed, the test indicated the occurrence of three potential problematic sequences of HKU2 sampled in 2006, which may represent the first sequences (among those included in the dataset) interested by the recombination event. Further attempts at dating have been performed on different subsets without including such problematic sequences, but also in those cases, datasets resulted in being not clock-like.

## 4. Discussion

Within the large group of viral pathogens infecting animals which comprise coronaviruses, SADS-CoV was the most recently discovered [12] and represents the etiological agent of several SADS outbreaks that occurred in Chinese swine herds between late 2016 and 2019 [16]. After the first peak of viral infection was recorded in the Chinese province of Guangdong in 2017, the expansion of the SADS-CoV outbreak was stopped, or at least slowed down, by the use of vaccines or other expedient measures [14]. In cases such as these, prevention and control of outbreaks can be relatively easy as the virus only spread within a limited geographic area (i.e., Fijian and Guangdong).

It is important to note that the viral infection of domestic animals not only causes economic problems, but can also be a serious threat to human health [32]. Coronaviruses are known to be harmful and heterogeneous viruses, able to infect a large number of hosts [33]. Furthermore, due to the recent SARS-CoV2 pandemic (see, i.e., [34]), the importance of a continuous molecular survey on coronavirus is being understood worldwide. Indeed, the ongoing SARS-CoV2 pandemic focused researchers’ attention on the crucial problem of cross-species transmission of viruses that originate from wildlife animal reservoirs, which represents a striking threat to human and animal health. This capability of transmission makes SADS-CoV a potential danger—not only for livestock, but also for human health—necessitating further research into its evolutionary history and origin. For this reason, since the onset of the first outbreaks, many holistic surveys are usually conducted in order to mitigate these infections [35].

Here, we performed the inference on the phylodynamics of SADS-CoV with the highest level of resolution possible to date using all the publicly available whole genomes whose sampling date were known on 21 June 2021. The evolutionary history of SADS-CoV in the wide context of the whole genus was depicted, also including in the analyses a large heterogeneous pool of *Alphacoronavirus*.

It has been established that both SADS-CoV and SARS-CoV have originated from the same genus of horseshoe bats (*Rhinolophus*). However, to the best of our knowledge, the direct progenitor of SADS-CoV remains still unknown. Consistent with previous researches, the phylogeny of *Alphacoronavirus*, here reconstructed based on both the whole genome and the gene S, confirms the close evolutionary relationship between SADS-CoV and the *Rhinolophus bat coronavirus HKU2*, as reported in previous studies (see, e.g., [12,13,14,36,37]). The short branches length of the SADS-CoV clade, as evidenced in the phylogenetic tree, indicates a rapid viral diversification; this may be explained by the need for the virus to adapt rapidly to new host. However, the phylogeny depicted in present study indicates that HKU2 (isolated for the first time in 2004) is not the direct progenitor of SADS-CoV, due to the position of SADS-CoV-related (also called HKU2-like) which may be the evolutionary intermediate step towards the present SADS-CoV. The phylogeny obtained based on both whole genome and gene S, shows that HKU2 is the ancestor of SADS-CoV, but it is not the closest one. This relationship is also confirmed by the phylogenetic tree obtained for the S gene. Indeed, in both trees, SADS-CoV displays high affinity with the SADS-CoV-related genomes (isolated between 2013 and 2016), illustrating a reciprocal monophyletic relationship. In addition, Pan et al. [12] and Zhou et al. [36] detected low levels of sequence identity for the gene S when comparing the HKU2 and SADS-CoV.

Phylogenetic reconstruction based on whole genomes places rodent CoV genomes externally to the main clade of the tree. This is not surprising, considering the global distribution of both rodents and rodent CoV. However, Yang et al. [16] found SADS-CoV to be phylogenetically close to rodent CoV. Likewise, our phylogenetic reconstruction based on the gene S depicted a close evolutionary path, with rodent CoV placed as a sister group of SADS-CoV, SADS-CoV-related, and HKU2. Conversely, Zhou et al. [36] performed whole genome phylogenetic analyses and found that rodent CoV is external to all alphacoronavirus genomes, while it is more internal within the phylogenetic tree based on ORF1a and ORF1b genes. Furthermore, consistent with findings from our research, Tsoleridis et al. [38] found that the phylogenetic position of rodent CoV and general topology of the tree varies depending on whether the gene or the whole genome was analysed.

These discrepancies in the phylogenetic position of rodent CoV when different molecular markers were analysed corroborate the recombination hypothesis advanced in previous researches [16,36], also considering that wild rats were frequently observed near farms in 2017 [16]. Indeed, although it is well known that horseshoe bats are reservoirs for HKU2-like CoVs, the transmission path from bats to pigs is still unclear. Yang et al. [16] hypothesised that faeces of bats infected with HKU2-like CoVs were accidentally introduced into pig farms by other animals, such as rodents, which are often found around pig farms. Indeed, viral transmission among species can lead to the development of new strains as a consequence of co-infection, and genetic recombination events may occur in intermediate hosts [39] due to the segmented nature of viral genomes, which contributes to the develop of a broad evolutionary diversity.

The results obtained by the attempt to molecular dating performed in the present study also corroborate the recombination hypothesis. Indeed, the failure of dating analysis, evidenced by the absence of a clock signal, is due to the lack of a positive association between sequence divergence and sampling dates. This very low correlation between genetic divergence and sampling dates has been also confirmed by TempEst analyses. Indeed, by exploring the temporal signal, the test indicated the occurrence of some factors disturbing the molecular clock, thus generating a not clock-likeness dataset (Rambaut et al., 2016). In addition, the test also pointed out the occurrence of three potential problematic sequences of HKU2 (identified as outliers) sampled in 2006, which may represent the first sequences interested by the recombination event. This finding may represent a limit to the reconstruction of the viral phylodynamics as, currently, it is not possible to perform a molecular dating excluding outliers. Indeed, the only use of genomes of SADS-CoV, which likely originated after the supposed recombination events, prevents dating analyses due to the limited number of genomes and the short sampling interval. In such a context, the genomes used in the present study were sampled between 2016 and 2019 with a homogeneous temporal distribution, with only one genome collected in 2016, 39 in 2017, six in 2018, and only one in 2019. Most of the used genomes show very similar nucleotide sequences, contributing to flatting the clock-likeness of the temporal signal. In the future, a large number of genomes, used to enlarge the sampling range and increase the variability, will be helpful to obtain the dating of the direct progenitor of SADS-CoV.

In accordance to our findings, Zhou et al. [36] suggested that SADS-CoV was introduced into pigs from bats once or multiple times, but subsequent genetic recombination disturbed the molecular clock. This disturbance created an obstacle in the search for the temporal origin of this virus.

Despite the lack of molecular dating, it may be hypothesized that the common ancestor to SADS-CoV predates the first outbreak, which occurred in August 2016 [13], by several months. Indeed, due to the nature of the typical symptoms of swine enteric coronaviruses, SADS-CoV may have passed through the farms unnoticed for several months, probably mistaken for a virus causing another enteric disease. Remarkably, SADS-CoV is more harmful than other porcine enteric coronaviruses; indeed, it caused the death of about 25,000 piglets in a short time [14], suggesting the need for a deep diagnostic survey intense activities that constantly allow its identification (see [12]).

One of the most important tasks during a viral outbreak is a continuous monitoring of genetic variation of the infective agent, in order to gain insights about the ongoing evolutionary dynamics of its spread. Currently, SADS-CoV is considered to be under control, but the re-emergence of infection in Guangdong in early 2019 [14] demonstrated that it could become dangerous again without warning. Considering how zoonotic infections originating in wildlife can become a significant threat to human health [19], a continuous monitoring plan for SADS-CoV is advisable, even during periods when the epidemic is apparently controlled. Indeed, constant monitoring would enable researchers to quickly identify new phases of infection. In a study aimed to evaluate human susceptibility to SADS-CoV cross-species transmission, Edwards et al. [40] demonstrated that SADS-CoV targets several host cell types from different species, and does not use any of the known coronavirus receptors for docking and entry to human cells. In addition, it is important to note that SADS-CoV originated in bats similar to other zoonotic viruses, including SARS-CoV and MERS-CoV, which infected intermediate hosts before passing to humans. In such a context, intensive farms, especially those with several potentially intermediate hosts, play a pivotal role in the spreading of viral human epidemics. Indeed, coronaviruses are very harmful pathogens for human health as the recombination and cross-species potential make their evolutionary path very difficult to be predictable, especially considering that recombination events between *Alphacoronavirus* and *Betacoronavirus* has already occurred (see [38]). All these conditions, together with the lack of knowledge on the direct progenitor and the potential for dispersal of *Alphacoronavirus*, make SADS-CoV a possible high-risk pathogen for humans, necessitating constant monitoring.

## Data Availability

Genomes and sequences analysed in the present study were taken from NCBI Virus database and are available at https://www.ncbi.nlm.nih.gov/labs/virus/vssi/#/, accessed on 21 June 2021.

## References

[1] Ismail M.M., Tang A.Y., Saif Y.M. (2003). Pathogenicity of turkey coronavirus in turkeys and chickens. Avian Dis..

[2] Cavanagh D. (2007). Coronavirus avian infectious bronchitis virus. Vet. Res..

[3] Tang Q., Song Y., Shi M., Cheng Y., Zhang W., Xia X.Q. (2015). Inferring the hosts of coronavirus using dual statistical models based on nucleotide composition. Sci. Rep..

[4] Cui J., Li F., Shi Z.L. (2019). Origin and evolution of pathogenic coronaviruses. Nat. Rev. Microbiol..

[5] Su S., Wong G., Shi W., Liu J., Lai A.C.K., Zhou J., Liu W., Bi Y., Gao G.F. (2016). Epidemiology, Genetic Recombination, and Pathogenesis of Coronaviruses. Trends Microbiol..

[6] Song Z., Xu Y., Bao L., Zhang L., Yu P., Qu Y., Zhu H., Zhao W., Han Y., Qin C. (2019). From SARS to MERS, Thrusting Coronaviruses into the Spotlight. Viruses.

[7] Woo P.C.Y., Lau S.K.P., Lam C.S.F., Lau C.C.Y., Tsang A.K.L., Lau J.H.N., Bai R., Teng J.L.L., Tsang C.C.C., Wang M. (2012). Discovery of Seven Novel Mammalian and Avian Coronaviruses in the Genus Deltacoronavirus Supports Bat Coronaviruses as the Gene Source of Alphacoronavirus and Betacoronavirus and Avian Coronaviruses as the Gene Source of Gammacoronavirus and Deltacoronavirus. J. Virol..

[8] Pratelli A., Colao V. (2015). Role of the lipid rafts in the life cycle of canine coronavirus. J. Gen. Virol..

[9] Gentles A.D., Guth S., Rozins C., Brook C.E. (2020). A review of mechanistic models of viral dynamics in bat reservoirs for zoonotic disease. Pathog. Glob. Health.

[10] Lau S.K.P., Woo P.C.Y., Yip C.C.Y., Fan R.Y.Y., Huang Y., Wang M., Guo R., Lam C.S.F., Tsang A.K.L., Lai K.K.Y. (2012). Isolation and Characterization of a Novel Betacoronavirus Subgroup A Coronavirus, Rabbit Coronavirus HKU14, from Domestic Rabbits. J. Virol..

[11] Gong L., Li J., Zhou Q., Xu Z., Chen L., Zhang Y., Cao Y. (2017). A new bat-HKU2–like coronavirus in swine, China, 2017. Emerg. Infect. Dis..

[12] Pan Y., Tian X., Qin P., Wang B., Zhao P., Yang Y., Wang L., Wang D., Song Y., Zhang X. (2017). Discovery of a novel swine enteric alphacoronavirus (SeACoV) in southern China. Vet. Microbiol..

[13] Zhou L., Sun Y., Lan T., Wu R.T., Chen J.W., Wu Z.X., Xie Q.M., Zhang X.B., Ma J.Y. (2018). Retrospective detection and phylogenetic analysis of swine acute diarrhoea syndrome coronavirus in pigs in southern China. Transbound. Emerg. Dis..

[14] Zhou L., Li Q.N., Su J.N., Chen G.H., Wu Z.X., Luo Y., Wu R.T., Sun Y., Lan T., Ma J.Y. (2019). The re-emerging of SADS-CoV infection in pig herds in Southern China. Transbound. Emerg. Dis..

[15] Li K., Li H., Bi Z., Gu J., Gong W., Luo S., Zhang F., Song D., Ye Y., Tanga Y. (2018). Complete Genome Sequence of a Novel Swine Acute Diarrhea Syndrome Coronavirus, CH/FJWT/2018, Isolated in Fujian, China, in 2018. Microbiol. Resour. Announc..

[16] Yang Y.L., Yu J.Q., Huang Y.W. (2020). Swine enteric alphacoronavirus (swine acute diarrhea syndrome coronavirus): An update three years after its discovery. Virus Res..

[17] Ji W., Wang W., Zhao X., Zai J. (2020). Cross-species transmission of the newly identifiedcoronavirus 2019-nCoV. J. Med. Virol..

[18] Latinne A., Hu B., Olival K.J., Zhu G., Zhang L., Li H., Chmura A.A., Field H.E., Zambrana-Torrelio C., Epstein J.H. (2020). Origin and cross-species transmission of bat coronaviruses in China. Nat. Commun..

[19] Daszak P., Epstein J.H., Kilpatrick A.M., Aguirre A.A., Karesh W.B., Cunningham A.A., Childs J.E., Mackenzie J.S., Richt J.A. (2007). Collaborative Research Approaches to the Role of Wildlife in Zoonotic Disease Emergence. Wildlife and Emerging Zoonotic Diseases: The Biology, Circumstances and Consequences of Cross-Species Transmission.

[20] Katoh K., Standley D.M. (2013). MAFFT Multiple sequence alignment software version 7: Improvements in performance and usability. Mol. Biol. Evol..

[21] Darriba D., Taboada G.L., Doallo R., Posada D. (2012). jModelTest 2: More models, new heuristics and parallel computing. Nat. Methods.

[22] Ronquist F., Teslenko M., Van der Mark P., Ayres D.L., Darling A., Höhna S., Larget B., Liu L., Suchard M.A., Huelsenbeck J.P. (2012). MrBayes 3.2: Efficient bayesian phylogenetic inference and model choice across a large model space. Syst. Biol..

[23] Gelman A., Rubin D.B. (1992). Inference from iterative simulation using multiple sequences. Stat. Sci..

[24] Scarpa F., Sanna D., Cossu P., Lai T., Curini-Galletti M., Casu M. (2018). A molecular approach to the reconstruction of the pre-Lessepsian fauna of the Isthmus of Suez: The case of the interstitial flatworm *Monocelis lineata sensu lato* (Platyhelminthes: Proseriata). J. Exp. Mar. Biol. Ecol..

[25] Drummond A.J., Rambaut A. (2007). BEAST: Bayesian evolutionary analysis by sampling trees. BMC Evol. Biol..

[26] Rambaut A., Drummond A.J., Xie D., Baele G., Suchard M.A. (2018). Posterior summarisation in Bayesian phylogenetics using Tracer 1.7. Syst. Biol..

[27] Kass R.E., Raftery A.E. (1995). Bayes factors. J. Am. Stat. Assoc..

[28] Suchard M.A., Weiss R.E., Sinsheimer J.S. (2001). Bayesian Selection of Continuous-Time Markov Chain Evolutionary Models. Mol. Biol. Evol..

[29] Rambaut A., Lam T.T., Carvalho L.M., Pybus O.G. (2016). Exploring the temporal structure of heterochronous sequences using TempEst. Virus Evol..

[30] Nguyen L.T., Schmidt H.A., von Haeseler A., Minh B.Q. (2015). IQ-TREE: A fast and effective stochastic algorithm for estimating maximum-likelihood phylogenies. Mol. Biol. Evol..

[31] Miller M.A., Pfeiffer W., Schwartz T. Creating the CIPRES Science Gateway for inference of large phylogenetic trees. Proceedings of the Gateway Computing Environments Workshop (GCE).

[32] Mackenzie J.S., Jeggo M. (2019). The One Health Approach—Why Is It So Important?. Trop. Med. Infect. Dis..

[33] Yu J., Qiao S., Guo R., Wang X. (2020). Cryo-EM structures of HKU2 and SADS-CoV spike glycoproteins provide insights into coronavirus evolution. Nat. Commun..

[34] Benvenuto D., Giovanetti M., Salemi M., Prosperi M., De Flora C., Alcantara L.C.J., Angeletti S., Ciccozzi M. (2020). The global spread of 2019-nCoV: A molecular evolutionary analysis. Pathog. Glob. Health.

[35] Wang Q., Vlasova A.N., Kenney S.P., Saif L.J. (2019). Emerging and re-emerging coronaviruses in pigs. Curr. Opin. Virol..

[36] Zhou P., Fan H., Lan T., Yang X.L., Shi W.F., Zhang W., Zhu Y., Zhang Y.W., Xie Q.M., Mani S. (2018). Fatal swine acute diarrhoea syndrome caused by an HKU2-related coronavirus of bat origin. Nature.

[37] Zhou P., Yang X.L., Wang X.G., Hu B., Zhang L., Zhang W., Si H.R., Zhu Y., Li B., Huang C.L. (2020). A pneumonia outbreak associated with a new coronavirus of probable bat origin. Nature.

[38] Tsoleridis T., Chappell J.G., Onianwa O., Marston D.A., Fooks A.R., Monchatre-Leroy E., Umhang G., Muller M.A., Drexler J.F., Drosten C. (2019). Shared common ancestry of rodent alphacoronaviruses sampled globally. Viruses.

[39] Coburn B.J., Wagner B.G., Blower S. (2009). Modeling influenza epidemics and pandemics: Insights into the future of swine flu (H1N1). BMC Med..

[40] Edwards C.E., Younta B.L., Grahama R.L., Leista S.R., Houa Y.J., Dinnon K.H., Simsc A.S., Swanstroma J., Gullyd K., Scobeya T.D. (2020). Swine acute diarrhea syndrome coronavirus replication in primary human cells reveals potential susceptibility to infection. Proc. Natl. Acad. Sci. USA.

